# Massive Upper Gastrointestinal Hemorrhage Secondary to Aortoenteric Fistula

**DOI:** 10.7759/cureus.98343

**Published:** 2025-12-02

**Authors:** Teresa Guimarães, Carolina Dias, Ana Pires, Gloria Campello

**Affiliations:** 1 Intensive Care Unit, Hospital Padre Américo, Centro Hospitalar do Tâmega e Sousa, Penafiel, PRT; 2 Internal Medicine, Centro Hospitalar do Tâmega e Sousa, Penafiel, PRT

**Keywords:** aortoenteric fistula, critical care, emergency medicine, hemodynamic shock, upper gastrointestinal hemorrhage

## Abstract

Aortoenteric fistula (AEF) is an uncommon condition that can present with severe gastrointestinal bleeding. We report the case of a 70-year-old woman with small-cell lung carcinoma and a known abdominal aortic aneurysm who arrived in hemorrhagic shock after massive hematemesis. CT angiography revealed direct communication between the aorta and the duodenum, consistent with an AEF. Despite resuscitation measures, broad-spectrum antibiotics, and coordination with vascular surgery, her clinical condition deteriorated rapidly prior to transfer for operative treatment. The patient died within hours. This case illustrates the rapidly progressive nature of AEF presentations and reinforces the need for early recognition and coordinated management.

## Introduction

Aortoenteric fistula (AEF) is an uncommon but potentially fatal condition defined as an abnormal communication between the aorta and the gastrointestinal tract. It may occur primarily due to erosion of an abdominal aortic aneurysm into the duodenum or secondarily following aortic surgery, particularly in the presence of vascular grafts [1-6].

Primary AEFs are extremely rare, occurring in less than 0.1% of abdominal aortic aneurysm cases, with only about 250 cases reported worldwide and an autopsy incidence of up to 0.07%. In contrast, secondary AEF, which develops after aortic repair, especially following graft implantation, is more common, with an incidence ranging from 0.36% to 1.6% after open aneurysm repair [6].

The pathophysiology of AEF involves the development of a pathological communication between the aorta and the gastrointestinal tract, most often the duodenum. Primary AEF usually results from erosion of an atherosclerotic abdominal aortic aneurysm or infection (septic aortitis), leading to destruction of both the aortic and adjacent intestinal walls with subsequent direct communication between the two structures [1-3]. The most frequent site is between the infrarenal aorta and the third portion of the duodenum, owing to their close anatomical relationship and retroperitoneal fixation [3]. Secondary AEF occurs after vascular surgery, particularly following aortic graft implantation. The predominant mechanism is mechanical or infectious erosion of the graft or suture line, which promotes chronic inflammation, pseudoaneurysm formation, and local infection, ultimately leading to tissue destruction and fistula formation [1,4-5].

Clinically, AEF presents with gastrointestinal bleeding, often preceded by a sentinel bleed, and may progress rapidly to hemorrhagic shock. Other features include abdominal pain, fever, or sepsis, particularly in the presence of graft infection. Diagnosis is challenging and requires a high index of suspicion. CT angiography (CTA) is the preferred diagnostic modality, although esophagogastroduodenoscopy and conventional angiography may provide additional information in selected cases [1,3,5]. Management constitutes a surgical emergency focused on hemorrhage control, hemodynamic stabilization, and infection management [7-9].

Given the rarity, high mortality, and diagnostic difficulty of AEF, reporting this case provides valuable insight into its clinical presentation and rapid deterioration, highlighting the importance of early recognition and intervention.

## Case presentation

**Figure 1:**
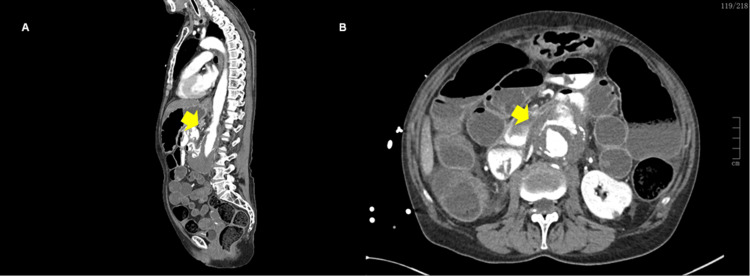
CTA demonstrating an AEF (A) Sagittal view showing extravasation of contrast from the abdominal aorta into the adjacent duodenum (yellow arrow). (B) Axial view confirming the communication between the aortic aneurysm and the duodenal lumen (yellow arrow). CTA: computed tomography angiography, AEF: aortoenteric fistula

A 70-year-old woman with small-cell lung carcinoma under surveillance after chemotherapy and a known abdominal aortic aneurysm under follow-up presented to the emergency department in hemorrhagic shock after sudden malaise, profuse hematemesis, and transient loss of consciousness. During pre-hospital care, she received tranexamic acid (1 g) and intravenous fluids. On arrival, she was hypotensive (BP 84/45 mmHg), tachycardic (HR 126 bpm), tachypneic, and exhibited peripheral hypoperfusion. After initial stabilization, a thorough clinical assessment was performed to identify features suggestive of upper gastrointestinal bleeding, such as hematemesis, melena, and a history of non-steroidal anti-inflammatory drug or anticoagulant use, or chronic liver disease. In this case, there was no history of melena or hematochezia. Oesophagogastroduodenoscopy was performed within the first hours after presentation, and CT was obtained approximately one hour after admission following hemodynamic stabilization. Laboratory results (Table 1) showed severe anemia (Hb 6.5 g/dL), with a previous hemoglobin of 9.2 g/dL, metabolic acidosis, high lactate levels (>15 mmol/L), and consumption coagulopathy. Urgent upper endoscopy revealed extensive gastric bleeding without an identifiable source, and CTA demonstrated a direct communication between the aortic lumen and the duodenal wall (Figure 1). Broad-spectrum antibiotics, corticosteroids, and transfusions were promptly initiated, and the patient was sedated, intubated, and mechanically ventilated. Administration included 2 g of ceftriaxone, 1 g of metronidazole, and 200 mg of hydrocortisone. In total, the patient received four units of packed red blood cells, 2000 units of prothrombin complex concentrate, and calcium gluconate. A vascular surgery team was urgently consulted, and transfer to a tertiary center was arranged. However, despite intensive resuscitation, the patient’s condition deteriorated rapidly, effectively preventing transfer. After multidisciplinary discussion and a family conference, care goals were redirected toward comfort measures, which included a morphine infusion and terminal extubation, and she died within hours of diagnosis.

**Table 1:** Blood tests results on hospital admission aPTT: activated partial thromboplastin time, INR: international normalized ratio, ALT: alanine transaminase, AST: aspartate transferase, GGTP: gamma-glutamyl transpeptidase, ALP: alkaline phosphatase

Test	Reference range	Results
White blood cells	4.5-11.0 x 10^3^/μL	8.80
Neutrophils	2.0-7.5 x 10^3^/μL	1.91
	40-70%	21.7
Lymphocytes	1.5-4.0 x 10^3^/μL	6.56
	20-45%	74.5
Monocytes	0.2-0.8 x 10^3^/μL	0.15
	5.0-12%	1.7
Eosinophils	0.04-0.40 x 10^3^/μL	0.14
	0.4-6.0%	1.6
Hemoglobin	12.0-15.0 x g/dL	6.5
Hematocrit	36.0-48.0%	21.0
Platelets	150-400 x 10^3^/μL	161
aPTT	25.4-36.9 seconds	40.7
Fibrinogen	200-400 mg/dL	188
INR	2-4% (therapeutic range)	1.24
Prothrombin time	9.4-12.5 seconds	14.5
Sodium	136-146 mmol/L	140
Potassium	3.50-5.10 mmol/L	4.8
Chloride	98-106 mmol/L	105
Magnesium	1.9-2.5 mg/dL	2.7
Urea	10-50 mg/dL	32
Creatinine	0.66-1.09 mg/dL	1.16
ALT	<31 UI/L	30
AST	<31 UI/L	15
Bilirubin	0.3-1.2 mg/dL	0.4
GGTP	7-32 UI/L	11
ALP	30-120 UI/L	113
Albumin	3.5-5.2 g/dL	3.1
C-reactive protein	<5 mg/L	39.5

## Discussion

AEF is a rare but life-threatening condition. Primary AEF most commonly affects elderly men with atherosclerotic abdominal aortic aneurysms but can also arise in the context of infectious aortitis, connective tissue disorders, vasculitis, or prior retroperitoneal malignancy or radiotherapy. Secondary AEF typically develops following open or endovascular aortic repair, particularly in the presence of synthetic grafts, graft infection, anastomotic pseudoaneurysms, emergency surgery, or repeated interventions. Reported incidence ranges from 0.36% to 1.6% after open repair and up to 3.9% in pseudoaneurysms treated with EVAR [1-6]. Certain medications, including systemic corticosteroids, immunosuppressants, chemotherapy, and antiplatelet or anticoagulant agents, may further increase the risk by impairing tissue integrity or exacerbating bleeding. At the same time, prolonged antibiotic exposure can predispose to graft infection [7-15].

Diagnosis of AEF remains challenging and requires a high index of suspicion. Clinically, patients often present with gastrointestinal bleeding, which may be preceded by a sentinel bleed, abdominal pain, fever, or sepsis, especially in the setting of graft infection. CTA is the diagnostic gold standard, while esophagogastroduodenoscopy and conventional angiography may provide additional information in selected cases. Early recognition is critical, as delayed diagnosis is associated with extremely high mortality [9-14].

Management constitutes a surgical emergency focused on hemorrhage control, hemodynamic stabilization, and infection management. Broad-spectrum antibiotics are indicated because of the direct communication between the aorta and the gastrointestinal tract, which can lead to severe infection and polymicrobial bacteremia. Surgical repair, either open or endovascular, remains the only definitive treatment, although outcomes are heavily influenced by comorbidities and hemodynamic status [16,17].

This case illustrates the typical clinical course of AEF and underscores the importance of early recognition and prompt intervention. The patient’s presentation with gastrointestinal bleeding and rapid deterioration reflects patterns described in the literature, reinforcing the need for vigilance in patients with a history of aortic aneurysm or repair. Reporting such cases adds to the understanding of AEF’s clinical spectrum and highlights the critical role of timely diagnosis and management.

## Conclusions

AEF is a rare but catastrophic cause of gastrointestinal hemorrhage that demands rapid recognition and coordinated multidisciplinary management. CTA remains the cornerstone of diagnosis, and prompt surgical involvement is essential for any chance of survival.

Despite advances in diagnostic and surgical techniques, prognosis remains extremely poor, particularly in patients with advanced comorbidities or hemodynamic instability. This case reinforces the importance of maintaining a high index of suspicion for AEF in patients with massive upper gastrointestinal bleeding and a known abdominal aortic aneurysm, ensuring early diagnosis and timely intervention whenever possible.
